# Phase Coupling Between Baroreflex Oscillations of Blood Pressure and Heart Rate Changes in 21-Day Dry Immersion

**DOI:** 10.3389/fphys.2020.00455

**Published:** 2020-05-21

**Authors:** Anatoly S. Borovik, Evgeniya A. Orlova, Elena S. Tomilovskaya, Olga S. Tarasova, Olga L. Vinogradova

**Affiliations:** ^1^State Research Center of the Russian Federation, Institute of Biomedical Problems, Russian Academy of Sciences, Moscow, Russia; ^2^Faculty of Biology, M.V. Lomonosov Moscow State University, Moscow, Russia; ^3^Faculty of Basic Medicine, M.V. Lomonosov Moscow State University, Moscow, Russia

**Keywords:** dry immersion, cardiovascular system, head-up tilt, baroreflex, heart rate, blood pressure, phase synchronization

## Abstract

**Introduction:**

Dry immersion (DI) is a ground-based experimental model which reproduces the effects of microgravity on the cardiovascular system and, therefore, can be used to study the mechanisms of post-flight orthostatic intolerance in cosmonauts. However, the effects of long-duration DI on cardiovascular system have not been studied yet. The aim of this work was to study the effects of 21-day DI on systemic hemodynamics and its baroreflex control at rest and during head-up tilt test (HUTT).

**Methods:**

Ten healthy young men were exposed to DI for 21 days. The day before, on the 7th, 14th, and 19th day of DI, as well as on the 1st and 5th days of recovery they were subjected to HUTT: 15 min in supine position and then 15 min of orthostasis (60°). ECG, arterial pressure, stroke volume and respiration rate were continuously recorded during the test. Phase synchronization index (PSI) of beat-to-beat mean arterial pressure (MAP) and heart rate (HR) in the frequency band of baroreflex waves (∼0.1 Hz) was used as a quantitative measure of baroreflex activity.

**Results:**

During DI, strong tachycardia and the reduction of stroke volume were observed both in supine position and during HUTT, these indicators did not recover on post-immersion day 5. In contrast, systolic arterial pressure and MAP decreased during HUTT on 14th day of DI, but then restored to pre-immersion values. Before DI and on day 5 of recovery, a transition from supine position to orthostasis was accompanied by an increase in PSI at the baroreflex frequency. However, PSI did not change in HUTT performed during DI and on post-immersion day 1. The amplitude of MAP oscillations at this frequency were increased by HUTT at all time points, while an increase of respective HR oscillations was absent during DI.

**Conclusion:**

21-day DI drastically changed the hemodynamic response to HUTT, while its effect on blood pressure was reduced between days 14 and 19, which speaks in favor of the adaptation to the conditions of DI. The lack of increase in phase synchronization of baroreflex MAP and HR oscillations during HUTT indicates disorders of baroreflex cardiac control during DI.

## Introduction

Long-duration exposure to microgravity is accompanied by profound changes in most of the physiological systems (Demontis et al., 2017), including disturbances in the sensorimotor, skeletal, and muscular systems, as well as changes in the regulation of cardiovascular system (LeBlanc et al., 2000; Eckberg, 2003). Obviously, such changes significantly limit the ability of cosmonauts to perform tasks after returning to the gravitational environment, which can significantly complicate their professional activity after landing not only on Earth, but also on the Moon (Rudas et al., 1999; Hughson et al., 2012).

Among the mechanisms for maintaining cardiovascular homeostasis after a long space flight, the leading role belongs to the reflexes from baroreceptors of the aortic arch and carotid sinuses (Benarroch, 2009). In addition, the reflexes from cardiopulmonary receptors play an important role in maintaining orthostatic tolerance (Convertino, 2014). Due to the activity of these reflexes, transition of human body from supine position to orthostasis is accompanied by regulatory changes in the cardiovascular system, which prevent blood pressure decrease, despite redistribution of blood in the body (Thrasher, 1994; Stewart, 2012). Under conditions of a long space flight, the baroreflex is impaired, which is one of the reasons for orthostatic intolerance after returning to Earth (Buckey et al., 1996; Waters et al., 2002).

To study the effects of microgravity on cardiovascular system, head-down bed rest (Kamiya et al., 2000; Grigoriev and Kozlovskaya, 2018) and dry immersion (Shul’zhenko and Will-Williams, 1976; Shulzhenko et al., 1976) are commonly used as ground-based models. Of note, the second model, in comparison with the first, better reproduces the effects of real space flight on most body systems (Tomilovskaya et al., 2019). Cardiovascular effects of dry immersion that last no more than 7 days are relatively well described (Iwase et al., 2000; Vinogradova et al., 2002; Navasiolava et al., 2011; de Abreu et al., 2017). In general, post-immersion changes in the cardiovascular system are similar to those observed after space flight. After 3 days of dry immersion, an increase in supine muscle sympathetic nerve activity (MSNA) was described along with unaltered HR and blood pressure (Iwase et al., 2000). In a later study, 3-day immersion without daily raise was followed by slight increases in supine heart rate (HR) and diastolic arterial pressure and a decrease in stroke volume (SV) (de Abreu et al., 2017). Importantly, the exposure to dry immersion environment dramatically changes all cardiovascular responses to orthostatic challenge: SV, blood pressure and HR shifts become significantly increased compared to their baseline values (Iwase et al., 2000; de Abreu et al., 2017), while no change in MSNA response to orthostasis is shown (Iwase et al., 2000). However, the effects of long-duration dry immersion on cardiovascular system and its regulation have not been studied yet.

The analysis of HR variability showed that autonomic cardiac control is readjusted during dry immersion toward predominance of the sympathetic mechanisms (Eshmanova et al., 2008). The sensitivity of the cardiac baroreflex estimated by the analysis of spontaneous HR and blood pressure fluctuations at the frequency of about 0.1 Hz (baroreflex waves; Julien, 2006; Stauss, 2007) was shown to decrease after dry immersion at supine position and an even more pronounced decrease is observed during head-up tilt test (de Abreu et al., 2017). It should be noted that when studying spontaneous oscillations of HR and blood pressure coordinated by the baroreflex, their amplitude characteristics are traditionally analyzed (Cooke et al., 1999; Akimoto et al., 2011; de Abreu et al., 2017). Noteworthy, the complex dynamics of physiological signals is also determined by their phase relationships. In this study, we introduce a novel approach to the assessment of baroreflex activity, based on the calculation of the phase relations of blood pressure and HR oscillations at the frequency of baroreflex waves using the phase synchronization index (PSI) (Borovik et al., 2014, 2019; Negulyaev et al., 2019). This method of analysis has several important advantages, since it provides more stable results than traditional methods (such as cross-spectral analysis) and does not require long continuous recording (Negulyaev et al., 2019). According to our previous data, PSI of blood pressure and HR in baroreflex frequency range increased significantly during orthostatic challenge in volunteers (Borovik et al., 2014) as well as during central hypovolemia induced by hemorrhage in laboratory rats (Negulyaev et al., 2019).

Orthostatic intolerance after dry immersion is associated with a decrease in MSNA, HR, and blood pressure (Iwase et al., 2000), as in vasovagal syncope which is clearly linked to the impaired baroreflex regulation of hemodynamics (Ogoh et al., 2004; Guasti et al., 2010; Schwartz et al., 2013b). Phase synchronization of baroreflex blood pressure and MSNA oscillations disappears in patients with vasovagal syncope during head-up tilt test a few minutes before the drop in blood pressure (Schwartz et al., 2013a). Regarding the phase synchronization of blood pressure and HR during syncope, we showed, for the first time, that the absence of PSI increase at an early stage of the head-up tilt test is associated with subsequent decompensation of hemodynamics and orthostatic intolerance (Borovik et al., 2019). These results suggest PSI of blood pressure and HR to be an informative measure of baroreflex activity in humans exposed to conditions of simulated or real microgravity.

Therefore, the aim of this work was to study the effects of long-duration (21-day) exposure to dry immersion on systemic hemodynamics and on the activity of a baroreflex (using PSI) during an orthostatic challenge (head-up tilt test).

## Methods

The study was conducted at dry immersion facilities of the Institute of Biomedical Problems, Russian Academy of Sciences (Tomilovskaya et al., 2019). The protocol of the study conforms to the Declaration of Helsinki and was approved by the Biomedical Ethics Committee of the Institute of Biomedical Problems, Russian Academy of Sciences (protocol N483 from 03.08.2018).

### Design of the Experiment

Ten healthy men (mean age 29.3 ± 3.8 years; height 176.4 ± 3.8 cm; weight 71 ± 10.6 kg; body mass index 22.7 ± 2.7) participated in the experiments. All subjects were familiarized with the protocol and informed of the risks associated with the experiment and gave their written consent to participate in the study. They were controlled by a medical team on duty during dry immersion exposure (21 days) as well as for 2 days before exposure and 2 days after its accomplishment. The beginning and the end of immersion both were at 8:45.

The experiment consisted of five stages. At each stage, two subjects were water immersed in two separate immersion baths. Water temperature in the bath was kept at 33 ± 1°C. Every evening (between 21:00 and 22:00), the subjects were lifted out of the bath for about 20 min for hygienic procedures. Our cardiovascular measurements (3 times during the immersion period – see Head-Up Tilt Test) were performed between 12:00 and 13:00 (before the lunch). For some studies, outside the scope of our experiment, the subjects were removed from the bath for a short time, during the measurements the subjects were in a supine position. In regular days (without orthostatic test) the time spent by the subjects outside the bath in the supine position was 15 ± 3 min, and in the sitting or standing position – 8 ± 2 min. In days with orthostatic tests these time intervals were extended to 57 ± 16 min and 22 ± 5 min respectively.

In the time free from procedures and measurements, the subjects could read, use the notebook or cellphone, watch TV, etc. Sleep time (light-off period) was 23:00–07:00. The total time spent by the subjects in the bath was 492 ± 2 h.

### Head-Up Tilt Test

Before the tilt test, the subject maintained supine position for 15 min. Then he was tilted to the 60° angle and the measurements were continued for another 16 min. In the head-up position the subject sat on the saddle, his legs hanging freely and not touching the support. At the request of the subject or with signs of the presyncope state, he was immediately returned to the supine position. Respiration frequency was constant during the test and controlled by the voice commands from the computer. Within the group of subjects, the respiratory rate ranged from 12 to 14 cycles per min. It was selected individually for each subject to be the most different from the frequency of baroreflex waves (∼0.1 Hz) but to remain comfortable for the subject.

During the experiment, ECG (PneumoCard, Medical computer systems, Russia), blood pressure and stroke volume (SV) (Finometer, Finapres Medical Systems, the Netherlands) and respiration frequency (nasal thermistor sensor) were continuously recorded. All signals were digitized at 1 kHz using E14-140 ADC (L-Card, Russia) and PowerGraph software (DISoft, Russia).

For each subjects, the measurements were performed six times: a day before the start of dry immersion (background measurement, B-1), on the 7th, 14th, and 19th days of the immersion (measurements DI7, DI14, and DI19, respectively) and also on days 1 and 5 after the end of immersion (during recovery period, measurements R1 and R5); R1 measurement was performed 28 h after the subject was lifted out of the bath. Such a schedule of measurements allowed us to study the time-course of cardiovascular changes during exposure to dry immersion and the recovery after cessation of the exposure.

### Data Processing

Data processing was performed *off-line* using home-made programs working under MATLAB (MathWorks Inc., United States). First, systolic, diastolic, pulse and mean arterial pressure (MAP) values as well as HR and SV values were determined for every cardiac cycle. Further calculations were performed in two 15 min intervals, the first of which preceded the head-up tilt (supine position), and the second began 1 min after changing the position of the body (orthostasis).

#### Calculation of Phase Synchronization Index

Baroreflex functioning was estimated by phase coupling of spontaneous MAP and HR oscillations in the frequency range of baroreflex waves (∼0.1 Hz), i.e., by the constancy of the difference in their phases. For this purpose, PSI of MAP and HR was calculated. The algorithm of PSI calculation was described in detail in our previous works (Borovik et al., 2014; Negulyaev et al., 2019). In brief, MAP and HR were resampled at 5 Hz using linear interpolation, then narrow-band signals were extracted by digital filtering from the obtained time series. Thereafter, the narrow-band MAP and HR signals were presented in the form of an analytic signal, that allowed us to determine their phases φ. For each frequency, normalized phase difference between HR and MAP was then calculated:

(1) $$\Delta \,\varphi =\left(\varphi_{H\,R}-\varphi_{M\,A\,P}\right)/2\,\pi \,m\,o\,d\,1$$

To quantitate the degree of phase synchronization at the certain frequency, PSI was obtained based on the calculation of Shannon entropy of Δφ distribution (Tass et al., 1998). Determined in this way, PSI is equal to 1 for “ideal” synchronization and is equal to zero for its complete absence. Using this algorithm, PSI values were obtained in the range from 0.02 to 0.5 Hz. Mean PSI in the band of interest (0.07–0.13 Hz) was calculated by averaging the respective values.

#### Spectral Analysis

Time series of MAP and HR were resampled at 5 Hz using linear interpolation. 102.4 s segments of equidistant time series (512 samples, half-overlapping from segment to segment) were then subjected to discrete fast Fourier transform to yield power spectra. Mean values of power spectral density (PSD) of MAP and HR in the baroreflex frequency band (from 0.07 to 0.13 Hz) were then calculated.

#### Statistical Analysis

Statistical data analysis was performed in GraphPad Prism 7.0 (GraphPad Software, La Jolla, CA, United States). The values are given as mean and SEM, besides anthropometric data and time intervals which are given as mean and SD. To estimate statistically significant differences Wilcoxon test was used. Statistical significance was reached at *p* < 0.05.

## Results

### The Effect of Dry Immersion on Hemodynamic Parameters During Orthostatic Challenge

**FIGURE 1:**
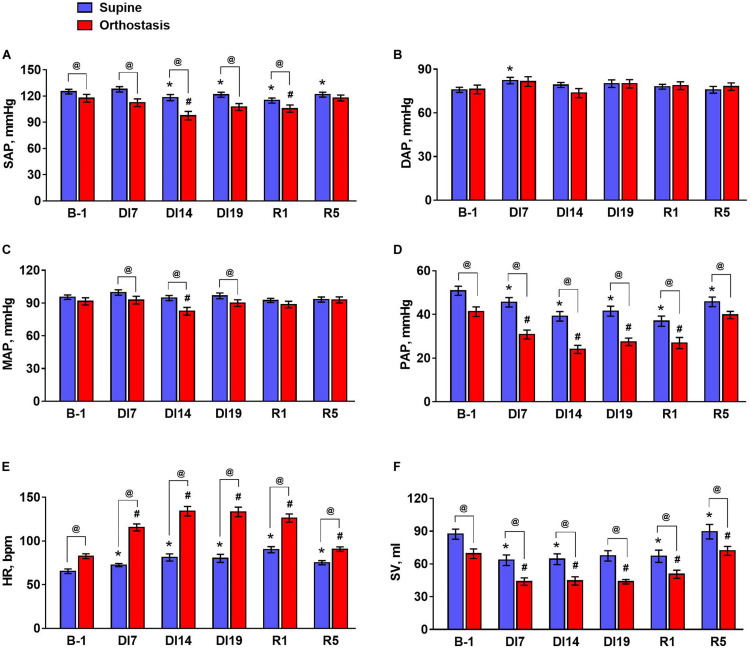
The values of hemodynamic parameters during tilt test 1 day before dry immersion (B-1), during dry immersion (DI7, DI14, and DI19) and during the recovery period (R1 and R5). **(A)** Systolic arterial pressure, SAP; **(B)** diastolic arterial pressure, DAP; **(C)** mean arterial pressure, MAP; **(D)** pulse arterial pressure, PAP; **(E)** heart rate, HR; **(F)** stroke volume, SV. Data are given for a group of 10 subjects (Mean ± SEM). *p < 0.05 compared with the value in supine position before immersion; ^#^*p* < 0.05 compared with the value in head-up position before immersion; ^@^*p* < 0.05 compared with the corresponding value in the supine position.

**FIGURE 2:**
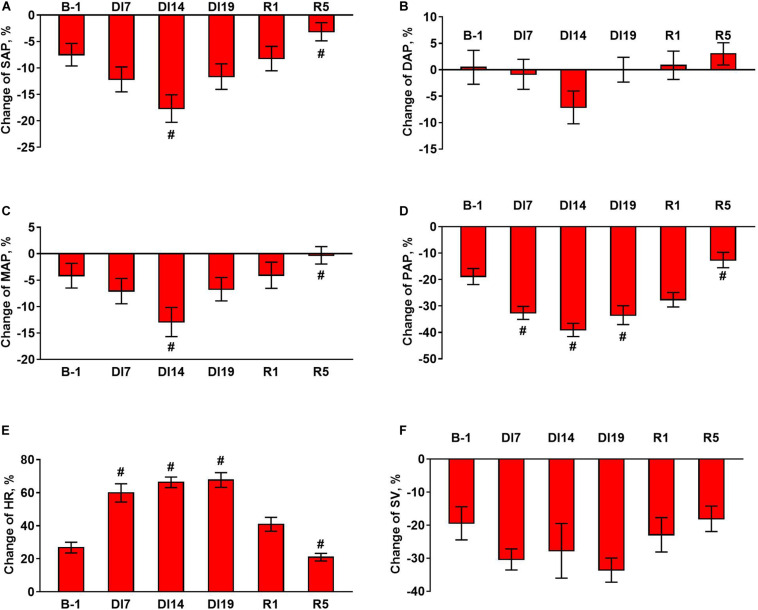
Changes in hemodynamic parameters during tilt test 1 day before dry immersion (B-1), during dry immersion (DI7, DI14, and DI19) and during the recovery period (R1 and R5). **(A)** Systolic arterial pressure, SAP; **(B)** diastolic arterial pressure, DAP; **(C)** mean arterial pressure, MAP; **(D)** pulse arterial pressure, PAP; **(E)** heart rate, HR; **(F)** stroke volume, SV. Data are given for a group of 10 subjects (Mean ± SEM). ^#^*p* < 0.05 compared with the value before immersion.

An exposure to dry immersion environment for 21 days led to significant changes in regulation of cardiovascular system, which were reflected in the values of hemodynamic parameters as well as in their responses to orthostatic challenge. In each subject, the mean values of hemodynamic parameters were calculated for a supine position and for orthostasis (Figure 1). In addition, the changes of the parameters on transition from the supine to the head-up position (as a percentage of background values) were calculated (Figure 2).

During the exposure to dry immersion, a slight decrease in systolic arterial pressure in the supine position was seen (Figure 1A). Diastolic pressure in the supine position was increased by 7 days of dry immersion and then did not differ from the baseline value (Figure 1B). Supine MAP did not change during the experiment (Figure 1C). In orthostasis, decreases in systolic (Figure 1A) and MAP (Figure 1C) were observed on day 14 of dry immersion. Accordingly, more pronounced responses of these indicators during orthostatic challenge were demonstrated on day 14 while on day 19 their responses did not change compared to pre-immersion values (Figures 2A,C). Pulse arterial pressure was the most affected by dry immersion: supine and orthostatic values of this indicator decreased (Figure 1D) and its response to orthostatic challenge increased (Figure 2D) starting from day 7 of the exposure. During the recovery, the systolic and pulse pressures in the supine position remained lower than before the immersion.

HR significantly increased during dry immersion in the supine position and especially at orthostasis (Figure 1E); the response of HR to the change of body position also increased significantly (Figure 2E). On the contrary, SV in supine position as well as at orthostasis decreased during dry immersion compared with the pre-immersion values (Figure 1F), although the percent reduction of SV in response to orthostatic challenge did not change (Figure 2F). The effects of dry immersion on HR and SV were prominently developed on the 7th day and were clearly seen even on the 5th day of recovery.

### The Effect of Dry Immersion on Phase Synchronization of MAP and HR During Orthostatic Challenge

**FIGURE 3:**
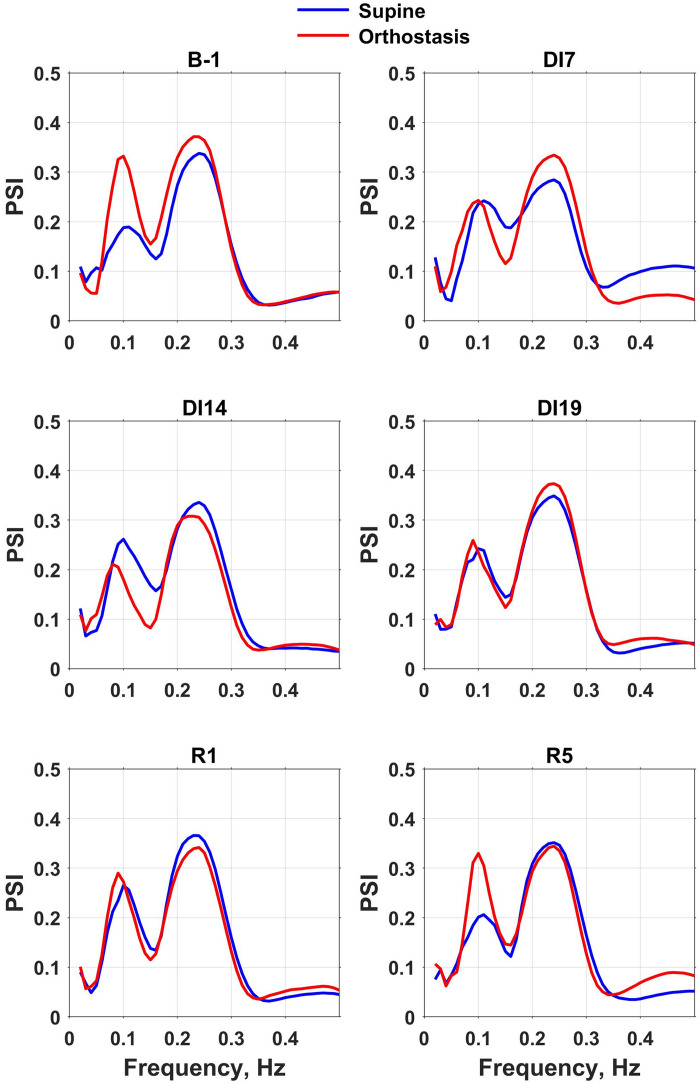
Phase synchronization index (PSI) spectra of mean blood pressure and heart rate calculated for the intervals corresponding to the supine and head-up position in a tilt test performed the day before dry immersion (B-1) during dry immersion (DI7, DI14, and DI19) and in the recovery period (R1 and R5). PSI spectra averaged over the group of 10 subjects are presented.

PSI spectra showed two distinct peaks in the frequency range studied (Figure 3). The high-frequency peak reflected the phase synchronization of MAP and HR at the respiration frequency. The amplitude of this peak did not depend on the body position during the tilt test and did not change during dry immersion and the recovery period. The frequency of this peak also did not change during the experiment, since the respiration rate was fixed (see Methods).

The position of the low-frequency peak corresponded to the frequency of baroreflex waves (about 0.1 Hz). Before the dry immersion, the change in body position from supine to orthostasis was followed by a significant increase of the low-frequency peak amplitude on PSI spectrum, which reflects an increase in the phase coupling of MAP and HR oscillations. However, during the exposure to dry immersion (on days 7, 14, and 19), the amplitude of the low-frequency peak on PSI spectrum did not change during head-up tilt test, which indicates a violation of the baroreflex control of HR. On the 5th day of recovery the spectra of PSI were very similar to respective spectra obtained before the dry immersion.

**FIGURE 4:**
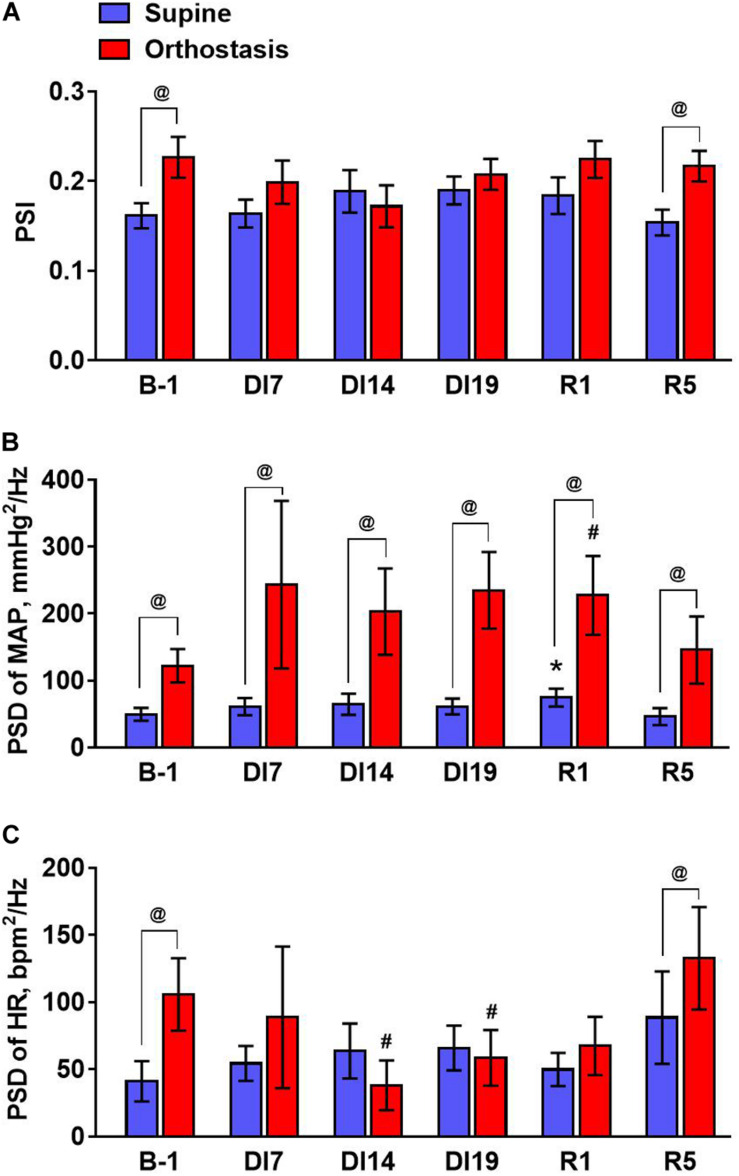
The values of the phase synchronization index of MAP and heart rate **(A)**, mean spectral density of MAP **(B)**, and heart rate oscillations **(C)** averaged in the baroreflex frequency range (from 0.07 to 0.13 Hz) during tilt test performed 1 day before dry immersion (B-1), during dry immersion (DI7, DI14, and DI19) and in the recovery period (R1 and R5). Data are given for a group of 10 subjects (Mean ± SEM). ^#^*p* < 0.05 compared with the value in orthostasis before immersion; ^@^*p* < 0.05 compared with the corresponding value in the supine position.

To quantify the effects of dry immersion on phase synchrony of MAP and HR, the average values of PSI were calculated in the frequency band from 0.07 to 0.13 Hz (Figure 4A). An increase in PSI in this frequency range during orthostasis was observed only in the tests performed before DI and on the 5th day of recovery. Importantly, oscillations of MAP in the frequency band from 0.07 to 0.13 Hz increased during the tilt test at all stages of the experiment – before and during dry immersion as well as during the recovery period (Figure 4B). However, oscillations of HR in this frequency band increased only in the tilt test performed before dry immersion and on the 5th day of recovery (Figure 4C). Moreover, on days 14 and 19 of the immersion, the power of HR fluctuations during orthostasis was reduced compared to the value before dry immersion.

## Discussion

Dry immersion is perhaps the most severe ground-based model of gravitational unloading. Up to now, reported studies focused on cardiovascular changes that occurred under the conditions of short-term (from 3 to 7 days) dry immersion (Navasiolava et al., 2011). Our work is the first comprehensive study of changes in cardiovascular system during a much longer (21 days) exposure to dry immersion. When planning the experiment, we were concerned that the subjects’ orthostatic tolerance would be severely deteriorated after such a long DI exposure. However, these concerns did not materialize. Tilt tests performed on days 7 and 14 of dry immersion were the most difficult for subjects according to their subjective assessment. On day 7 one of the subjects had signs of a pre-syncope condition during orthostasis and on day 14 of the immersion two cases of a pre-syncope condition were recorded (the measurements were canceled ahead of schedule). However, on day 19 head-up tilt test was better tolerated according to both subjective sensations, which correlated with the reduced effects of dry immersion on some cardiovascular indicators between day 14 and day 19. This observation can reflect the adaptation of cardiovascular system and the body as a whole to the conditions of dry immersion.

We conducted detailed studies of hemodynamic changes caused by prolonged exposure to DI, both at rest (in a supine position) and during orthostatic challenge (passive head-up tilt test). It was shown that the levels of systolic and MAP decreased during orthostasis on day 14 of dry immersion, but did not change on day 19. Along with that, HR during dry immersion was increased even at rest and increased sharply in response to the transition of body to head-up position. This effect was observed already on the 7th day and practically did not change with further increase of dry immersion duration. Therefore, the data obtained indicate that the pattern of hemodynamic changes associated with a prolonged exposure to dry immersion is similar to that observed with shorter exposure (Navasiolava et al., 2011). However, we showed for the first time that major changes in hemodynamic parameters develop by 7–14 days of immersion and then stabilize. Importantly, the dynamics of cardiovascular system shifts during dry immersion is similar to that in space flight: severe alterations at the beginning of the flight subside or stabilize at later stages (Hughson et al., 2012; Eckberg et al., 2016). It can also be noted that even after prolonged DI, hemodynamic parameters almost return to their initial values as soon as on the 5th day of recovery.

To evaluate the changes in the cardiac baroreflex during simulated gravitational unloading, we used a novel approach based on estimation of the phase relations of BP and HR fluctuations in the frequency range of baroreflex waves (Borovik et al., 2014, 2019; Negulyaev et al., 2019). Importantly, the observed changes in cardiac baroreflex sensitivity during orthostasis depend on the method of assessing baroreflex activity. Using the neck suction technique, an increase in baroreflex sensitivity during transition to head-up position (tilt test) has been shown (Ogoh et al., 2003; Akimoto et al., 2011). However, using the sequence method, a decrease in the sensitivity of the baroreflex was shown (Akimoto et al., 2011; Silvani et al., 2017). A similar decrease in the sensitivity of cardiac baroreflex during tilt test was revealed using cross-spectral analysis: a decrease in the amplitude of the transfer function between blood pressure and heart rate in the range of baroreflex waves (Cooke et al., 1999; Akimoto et al., 2011). Of note, the methods which are commonly used for assessing baroreflex activity in physiological experiments and in medical practice are based on recording changes in HR with changes in BP. In our method, as well as in cross-spectral analysis, spontaneous fluctuations of BP and HR are studied, but PSI is used as a quantitative measure of the phase coupling of these fluctuations (Tass et al., 1998) showing the degree of their synchronization in a certain frequency range. We have shown that the value of the PSI of BP and BP oscillations at the frequency of baroreflex waves correlated well with the coherence calculated by the cross-spectral method (Negulyaev et al., 2019).

It should be noted that, in addition to baroreflex activity, diverse factors related to the vital activity of the body and external influences affect vascular tone and heart function. Such influences induce non-periodic changes in hemodynamic parameters and can mask the relatively regular respiratory and baroreflex waves of blood pressure and heart rate. We showed that PSI is less affected by random fluctuations in hemodynamic parameters than coherence and gain of spontaneous cardiac baroreflex (Negulyaev et al., 2019) and, therefore, can be used to characterize the activity of cardiac baroreflex when analyzing the relatively “noisy” time series. In contrast to the decrease in the amplitude of the transfer function between blood pressure and heart rate in the frequency band of baroreflex waves (Cooke et al., 1999; Akimoto et al., 2011), the phase coupling of BP and HR oscillations at the baroreflex frequency was augmented in our study during orthostasis. Using cross-correlation analysis, Silvani et al. (2017) also showed an increase in the coupling of blood pressure and heart rate fluctuations when the body position was changed from horizontal to vertical. Based on the analysis of changes in heart rate variability indicators concomitant with orthostasis, the authors made a bold conclusion that changes in baroreflex sensitivity are associated with a decrease in vagal effects on the heart, and increased coupling of blood pressure and heart rate fluctuations, at least in part, is associated with increased fluctuations in vascular resistance in a head-up position (Silvani et al., 2017).

Starting from Ekberg’s classical studies, it is known that the baroreflex sensitivity in space flight and in the ground-based simulations of gravitational unloading decreases or remains unchanged (Fritsch et al., 1992; Eckberg, 2003; Ferretti et al., 2009; Hughson et al., 2012; de Abreu et al., 2017). Our data on the ceased effect of orthostasis on PSI of blood pressure and HR during the whole 21-day exposure to dry immersion are in accordance with these earlier reports. On the 5th day of the recovery period, the baroreflex control of HR was restored: the similar changes in PSI spectrum were observed during tilt test, as before the start of dry immersion. Our data suggest that impaired phase coupling of blood pressure and heart rate during simulated microgravity was not associated with reduced amplitude of oscillations in vascular resistance in a head-up position, which is supported by the data on preserved control of MSNA in dry immersion environment (Iwase et al., 2000). However, the transition of blood pressure oscillations into HR oscillations was greatly disturbed by dry immersion. Therefore, our studies have shown that long-duration exposure to dry immersion results in prominent changes of heart rate baroreflex control.

Along with reporting novel observations our study has several limitations. First, the dynamics of PSI of blood pressure and HR during post-immersion period should have been studied in more detail, which would indicate the day on which the restoration of baroreflex control after long-duration dry immersion occurs. The second limitation is the relatively small number of the dry immersion experiment participants, in future the observed data need to be confirmed on a larger number of subjects. The next one is lumbar pain which occurs during exposure to dry immersion (de Abreu et al., 2017) and therefore could influence the results of our orthostatic tests. To control this factor, the participants were asked daily to evaluate lumbar pain using a subjective 10-level score. Importantly, lumbar pain was reported by the participants during days 3–5 of immersion, but not on day 6 and later. Therefore, lumbar pain could not affect the results of orthostatic tests, which were performed starting from immersion day 7.

## Conclusion

In this work, for the first time, the changes in hemodynamic parameters were studied during a prolonged (21 days) exposure to dry immersion environment, ground-based model of the effects of microgravity. It was shown that changes in hemodynamic parameters in response to orthostatic challenge are most pronounced on days 7–14 of dry immersion. Violation of baroreflex control was detected already on the 7th day of exposure to dry immersion, persisted during the entire period of exposure and in the early recovery period (1st day). It should be noted that the absence of changes in the PSI in the baroreflex frequency range with a change in body position in the tilt test is typical for patients with fainting of a vasovagal nature (Borovik et al., 2019). Thus, our novel method can be used for assessing orthostasis-induced changes in baroreflex activity in the conditions of microgravity and in the development of countermeasures aimed at preventing orthostatic intolerance that occurs in cosmonauts/astronauts when they return to Earth’s gravity.

## Data Availability Statement

The datasets generated for this study are available on request to the corresponding author.

## Ethics Statement

All the procedures were conducted in accordance with the Declaration of Helsinki and were approved by the Biomedical Ethics Committee of the Institute of Biomedical Problems, Russian Academy of Sciences (protocol N483 from 03.08.2018). All potential risks were explained to the participants, and they gave written informed consent to participate in the experiment. The participants were controlled by a medical team on 24 h duty during dry immersion exposure, 2 days before the dry immersion and 2 post-immersion days. The participants were provided by accident insurance during the experiment.

## Author Contributions

AB, EO, ET, and OV conceived and designed the study. AB and EO were involved in the acquisition of the data. AB, EO, and OT analyzed cardiovascular data and performed the statistical analysis. AB, EO, and OT drafted the manuscript. AB, ET, and OV revised the manuscript critically. All authors given final approval of the version to be submitted.

## Conflict of Interest

The authors declare that the research was conducted in the absence of any commercial or financial relationships that could be construed as a potential conflict of interest.
